# Immunocompromisation of wheat host by L-BSO and 2,4-DPA induces susceptibility to the fungal pathogen *Fusarium oxysporum*

**DOI:** 10.1007/s44154-023-00137-7

**Published:** 2024-04-09

**Authors:** Abhaya Kumar Sahu, Punam Kumari, Bhabatosh Mittra

**Affiliations:** 1 P.G. Department of Biosciences and Biotechnology, Fakir Mohan University, VyasaVihar, Balasore, Odisha 756089 India; 2 MITS School of Biotechnology, Bhubaneswar, Odisha 751024 India

**Keywords:** Susceptibility, *Triticum aestivum*, *Fusarium oxysporum*, BSO, 2,4-DPA, γ-ECS, PAL

## Abstract

Susceptibility is defined as the disruption of host defence systems that promotes infection or limits pathogenicity. Glutathione (GSH) is a major component of defence signalling pathways that maintain redox status and is synthesised by γ-glutamyl cysteine synthetase (γ-ECS). On the other hand, lignin acts as a barrier in the primary cell wall of vascular bundles (VBs) synthesised by phenylalanine ammonia-lyase (PAL) in the intracellular system of plants. In this study, we used two inhibitors, such as L-Buthionine-sulfoximine (BSO), which irreversibly inhibits γ-ECS, and 2,4-dichlorophenoxyacetic acid (DPA), which reduces PAL activity and leads to the induction of oxidative stress in wheat (*Triticum aestivum*) seedlings after exposure to *Fusarium oxysporum*. Seedlings treated with 1 mM L-BSO and 2,4-DPA showed high levels of hydrogen peroxide (H_2_O_2_), malondialdehyde (MDA), carbonyl (CO) content, and low activity of antioxidative enzymes [superoxide dismutase (SOD), catalase (CAT), ascorbate peroxidase (APX), and glutathione reductase (GR)] as compared to wild-type (WT) seedlings under *F. oxysporum* infection. Further, the content of reduced glutathione (RGSH), ascorbate (ASC), and lignin was decreased in BSO and DPA treated seedlings as compared to WT seedlings during *Fusarium* infection. Moreover, treatment with BSO and DPA significantly inhibited the relative activity of γ-ECS and PAL (*P* ≤ 0.001) in WT seedlings during *Fusarium* infection, which led to disintegrated VBs and, finally, cell death. Our results demonstrate that inhibition of γ-ECS and PAL by BSO and DPA, respectively, disrupts the defence mechanisms of wheat seedlings and induces susceptibility to *F. oxysporum*.

**Supplementary Information:**

The online version contains supplementary material available at 10.1007/s44154-023-00137-7.

## Introduction

Wheat (*Triticum aestivum* L.) is one of the globe’s largest economically valuable poaceae crops (Bahadur et al. 2022), and its production is severely affected by a devastating soil-borne fungus, *Fusarium oxysporum* (Sampaio et al. 2020). The spores of *F. oxysporum* species can penetrate the host through wounds in the root region. The penetration process is increased by certain hydrolysing enzymes secreted by *Fusarium* (Gabrekiristos and Demiyo 2020). Inside the root, the cortex is occupied by young mycelia, which invade the endodermis and finally enter the xylem vessels through the pits. The xylem vessels are blocked due to mycelia producing microconidia in the entire shoot, consequently lowering the transpiration rate, which leads to significant cytological alterations resulting in vascular wilt, corm rot, root rot, and damping-off diseases (Garcés-Fiallos et al. 2017). In addition, *Fusarium* produced mycotoxins, including type B trichotecenes, deoxynivalenol (DON), nivalenol (NIV), 3-acetyl and 15-acetyl deoxynivalenol (3ADON and 15ADON), and 4-acetyl nivalenol (4ANIV), damaged the host tissues (Gabrekiristos and Demiyo 2020). This induces wilting of the entire plant, which eventually leads to death (Abou El-ghit HM 2016). The disturbed metabolism in chloroplasts and mitochondria leads to the overproduction of reactive species (RS), reactive halogen species (RHS), reactive nitrogen species (RNS), and reactive oxygen species (ROS) such as hydroxyl (OH•), hydrogen peroxide (H_2_O_2_), superoxide (O_2_•^−^), singlet oxygen (^1^O_2_), nitric oxide (NO•), HOBr, hypochlorous acid (HOCl), and HOI, which results in oxidative stress (Pisoschi et al. 2021). This RS causes damage to critical biological components such as nucleic acids, proteins, and lipids. ROS can oxidise polyunsaturated fatty acids (PUFAs), resulting in the generation of lipid oxidation products such as MDA, 4-oxo-2-nonenal (4-HNE), and acrolein (ACR), known as reactive carbonyl species (RCS), and also producing carbonyl groups (CO) on proteins by irreversible reaction (Vishnu et al. 2021). To control RS levels, oxidative damage, and the redox status of the cell, plants manufacture a diversity of enzymatic (CAT, SOD, APX) and non-enzymatic antioxidants (GSH, ASC, β-carotene, α-tocopherol) (Hasanuzzaman et al. 2020).

To neutralise the ROS induced oxidative stress inside the vascular system (VS), the plants have developed a basal defence system constitutively through the induction of lignin metabolic pathways. Lignin is a prime phenolic compound that organizes the secondary cell wall in vascularized plants and is anabolized from L-phenylalanine (L-Phe) and cinnamate by committed PAL (Feduraev et al. 2021). Similarly, the O_2_^•−^ and H_2_O_2_ radicals reinforce the cell wall by lignification and inhibit the ingression of microbial proliferation in VBs (Xie et al. 2018). The host starts a non-enzymatic antioxidant GSH signalling pathway to regulate the ROS and RNS during pathogen infestation (Juan et al. 2021). It is generated by using γ-ECS and glutathione synthetase (GS) enzymes and has two forms, such as reduced glutathione (RGSH) and oxidized glutathione (GSSG) (Chen et al. 2020). It is an essential metabolite for maintaining and controlling cellular redox status and providing immunity to pathogen infection in plants (Jelena et al. 2021) through the ASC-GSH cycle (Datta and Chattopadhyay 2018).

Evidence suggests that pathogen invasion and susceptibility are facilitated by host plant defence enzyme inactivation (Peyraud et al. 2017). For example, the suppression of guaiacol-peroxidase (GPx) and polyphenol oxidase (PPO) activities by potassium cyanide (KCN) induced susceptibility to *F. graminearum* infection in wheat (Mohammadi and Kazemi 2002). Coronatine is a toxic compound that analogs the function of the hormone jasmonic acid-isoleucine, which inhibits salicylic acid cascade pathways, promotes systemic susceptibility, and causes disease symptoms in plants (Zheng et al. 2012). GPx is recognised as the chief enzyme in the cellular system for the detoxification of peroxide and provides protection against ROS induced oxidation. The GPx activity was inhibited by methylmercury (MeHg) and rendered mouse brain cells susceptible to oxidative stress (Farina et al. 2011). However, GPx activity was also inhibited by NO, homocysteine, and mercaptans (Lubos et al. 2011). In addition, the competitive inhibitors of the PAL enzyme, such as (S)-2-aminooxy-3-phenylpropionic acid (AOPP), piperonylic acid (PIP), 2-aminoindane-2-phosphonic acid (AIP), 3,4-methylenedioxycinnamic acid (MDCA), and O-benzylhydroxylamine (OBHA), also significantly reduce the level of intermediates of the phenylpropanoid pathway in *Lycopersicon esculentum* (Tyagi et al. 2022). It is also reported that suppression of PAL activity induces susceptibility to fungal pathogens in wheat, tobacco, and flax plants (Lee et al. 2018). Moreover, the inactivation of the γ-ECS enzyme, which possesses a low level of GSH, resulted in an immunocompromised Arabidopsis mutant against pathogens (Hossain et al. 2022). It is also reported that a lethal embryo was produced due to a lack of the γ-ECS gene (Noctor et al. 2018). Furthermore, L-Buthionine-sulfoximine (L-BSO) preferentially inhibits γ-ECS, an enzyme that works as a rate-limiting catalyst in the manufacture of GSH in cancerous cell lines (Noctor et al. 2012; Wang et al. 2015) and carrot plants (Flores-Cáceres et al. 2015).

Hence, previous studies suggested that GSH and lignin play a crucial role in pathogen resistance. Application of inhibitors of γ-ECS and PAL might compromise plant health. Therefore, in this study, we applied this concept by using two inhibitors, L-BSO and DPA of γ-ECS and PAL, respectively, and assessed the induction of susceptibility in wheat seedlings against *F*. *oxysporum*.

## Results

### Reduction of plant growth and induction of oxidative stress by BSO and DPA treatment

**Fig. 1:**
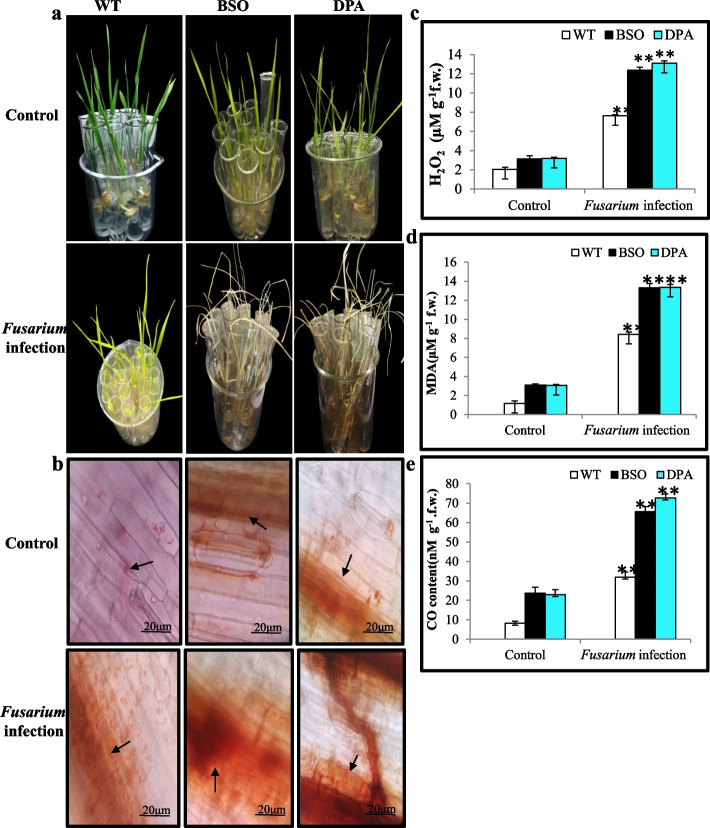
Effect of inhibitors on the generation of oxidant content during *Fusarium* infection. **a** The upper  panel shows the phenotype of WT, BSO, and DPA treated seedlings under non-stress and *Fusarium* infection condition. **b** The bottom panel shows the response of histochemical assessment of H_2_O_2_ in WT, BSO, and DPA treated leaf tissues under non-stress and *Fusarium* infection condition. **c** Changes in the level of H_2_O_2_ content in shoot tissues. **d** Lipid peroxidation expressed in terms of MDA content in shoot tissues. **e** Changes in the level of CO content in shoot tissues. *, ** denote significance at *p* ≤ 0.05 and *p* ≤ 0.001, respectively

The wild-type (WT) seedlings showed better growth under *Fusarium* stress as compared to the BSO and DPA treated seedlings,where the leaves exhibited signs of yellowing and wilting (Fig. 1a). A significant reduction in shoot length (SL, by 33.3% and 27.46%) and root length (RL, by 15.80% and 8.82%) was also observed in BSO and DPA-treated seedlings as compared to WT under *Fusarium* infection. Similarly, the fresh weight (FW, by 16.96% and 20.58%), dry weight (DW, by 31.26% and 29.33%), and relative water content (RWC, by 57.97% and 54.86%) were reduced in BSO and DPA treated seedlings as compared to WT under *Fusarium* infection (Table 1).

**Table 1:** Effect of inhibitors on growth and development of WT, BSO, and DPA treated seedlings under *Fusarium* infection

Treatment	SL(cm)	RL(cm)	FW(mg/seedling)	DW(mg/seedling)	RWC(%)
WT	15.77 ± 2.41	9.25 ± 1.13	128.52 ± 10.83	21.39 ± 6.00	92.52 ± 23.42
BSO	9.98 ± 1.85	8.04 ± 0.62	96.40 ± 30.39	12.80 ± 4.17	85.48 ± 12.10
DPA	9.85 ± 0.97	7.08 ± 0.92	111.21 ± 13.63	11.78 ± 4.03	84.32 ± 18.81
*Fus*	8.63 ± 1.24*	5.44 ± 0.61**	87.30 ± 4.58	8.278 ± 4.74*	81.24 ± 15.76**
BSO + *Fus*	5.75 ± 2.08	4.58 ± 0.54	72.49 ± 3.37*	5.69 ± 3.67	34.14 ± 12.21
DPA + *Fus*	6.26 ± 1.52*	4.96 ± 0.88*	69.33 ± 6.17	5.85 ± 3.25*	36.67 ± 8.13*

Data represent mean value ± SE from three replicates, *n* = 5

WT: (wild type) seedlings, *Fus:* WT seedlings infected with *Fusarium*, BSO + *Fus:* BSO treated seedlings infected with *Fusarium*, DPA + *Fus:* DPA treated seedlings infected with *Fusarium*

^*^ and ** represent significant and highly significant differences as compared to WT at *p* ≤ 0.05 and *p* ≤ 0.01 respectively

The H_2_O_2_ content, an indicator of oxidative stress due to an imbalance of the redox system, was estimated in WT, BSO, and DPA exposed to *Fusarium* infection. No significant visual difference was seen in the accumulation of H_2_O_2_ in WT, BSO, and DPA under non-stress condition. The reddish brown spots, indicative of H_2_O_2_ accumulation, were increased in the leaf tissues of BSO and DPA-treated seedlings but less in WT during infection (Fig. 1b). These results were corroborated by measuring the H_2_O_2_ content in the shoot tissues. The H_2_O_2_ level was enhanced by ~1.75 to ~1.82-fold in BSO and DPA-treated seedlings as compared to WT when exposed to *Fusarium-induced* stress (Fig. 1c). The lipid peroxidation product MDA has been used as an oxidative stress marker in plant responses. The MDA was observed to be ~1.58 to ~1.60-fold greater in BSO and DPA than WT during infection (Fig. 1d). Moreover, the protein carbonyl content (CO) is indicative of a stress marker in susceptible plants. The CO content in BSO and DPA-treated seedlings was increased by ~2.16 and ~2.33-fold as compared to WT (Fig. 1e).

### Treatment with BSO and DPA reduce the ROS scavenger’s activity

**Fig. 2:**
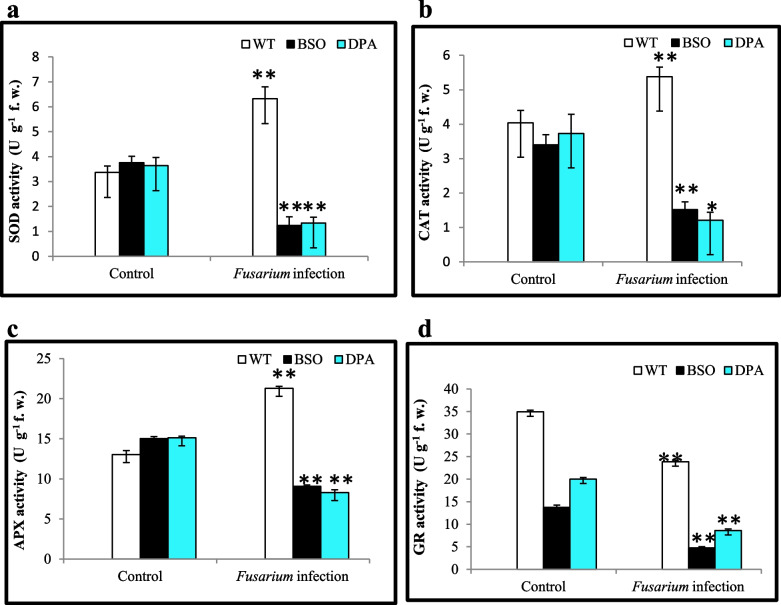
Influence of inhibitors on antioxidant enzyme machinery in WT, BSO, and DPA treated shoot tissues after *Fusarium* infection. **a** Changes in the activity of SOD, **b** Changes in the activity of CAT, **c** Changes in the activity of APX, **d** Changes in the activity of GR. *, ** denote significance at *p* ≤ 0.05 and *p* ≤ 0.001, respectively

In order to detoxify ROS, plants have a dynamic antioxidant machinery that is required for reducing ROS under biotic stresses. The effect of BSO and DPA on the activity of antioxidant enzymes that assist in quenching ROS, SOD, CAT, and APX was also measured in shoot tissues. All of these enzymes’ activities were reduced ~2.0 to ~3.0- fold as compared to WT when exposed to *Fusarium* infection. No significant difference was seen in the antioxidant activity of WT, BSO, and DPA under non-stress condition (Fig. 2a, b, and c). The GR activity was also decreased ~5.0-fold in BSO and DPA-treated shoot tissues under infection condition (Fig. 2d). Overall, supplementation with BSO and DPA reduced antioxidant enzyme activity in WT under the *Fusarium* infection condition more than control.

### Effects of inhibitors on ASC-GSH cycle and lignin accumulation

**Fig. 3:**
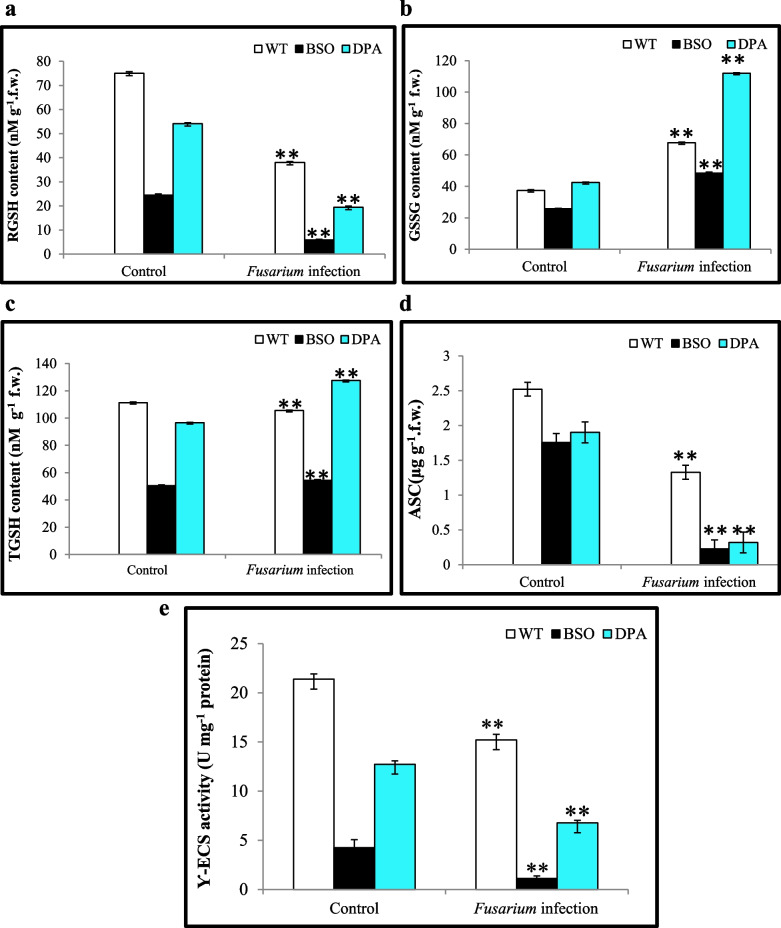
Effect of inhibitors on antioxidant content and Ƴ-ECS activity in WT, BSO, and DPA treated shoot tissues under *Fusarium* stress. **a** RGSH content, **b** GSSG, **c** TGSH, **d** ASC, and **e** Ƴ-ECS activity. *, ** denote significance at *p* ≤ 0.05 and *p* ≤ 0.001, respectively

In the ASC-GSH cycle, RGSH regenerates the oxidised ASC through the detoxification of H_2_O_2_ and MDA, providing defense responses in plants. The RGSH content was decreased ~5.42-fold in BSO and ~2.0-fold in DPA treated tissues than in WT shoot tissues under *Fusarium* infection (Fig. 3a). Moreover, the GSSG content was decreased ~1.66-fold in BSO and increased ~1.57-fold in DPA treated shoot tissues under infection condition (Fig. 3b). Overall, the total glutathione (TGSH) content was reduced ~2.1-fold in BSO and enhanced ~1.2-fold in DPA shoot tissues under infected condition (Fig. 3c). Another ROS quencher, ASC content, was reduced ~7.5-fold in both BSO and DPA treated shoot tissues when infected (Fig. 3d). Additionally, the γ-ECS enzyme replenishes the GSH pool to help plants defend themselves from infection. The γ-ECS activity was reduced ~8.2-fold in BSO and ~2.6-fold in DPA treated shoot tissues, respectively, as compared to WT during infection (Fig. 3e). Hence, the BSO inhibited the defense system through inactivation of γ-ECS which result in low level of RGSH, GSSG and TGSH content as compared to WT and DPA plants under both control and infection condition.

**Fig. 4:**
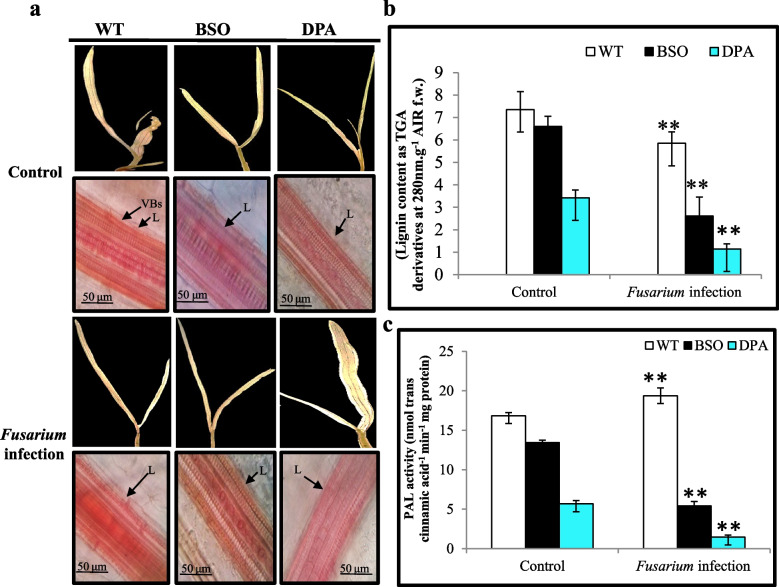
Effect of inhibitors on Lignin content and PAL activity in WT, BSO, and DPA treated shoot tissues under *Fusarium* stress. **a** Lignin accumulation, **b** Lignin content, **c** PAL activity. VBs-Vascular bundles, L-Lignin* and, ** denote significance at *p* ≤ 0.05 and *p* ≤ 0.001, respectively

Lignin is a polymer of phenolic compounds that is embedded in the VBs to restrict the fungal invasion in plants. The lignin accumulation in plants was assayed (indicated as red spots), which decreased in the VBs of BSO and DPA treated shoot tissues compared to WT. There is no significant difference in the VBs of WT, BSO, and DPA treated shoot tissues under control condition (Fig. 4a). Similarly, the BSO and DPA treated shoot tissues showed ~2.14 and ~5.27-fold lower lignin content than WT shoot tissues under infection (Fig. 4b). The lignin precursor synthesizing enzyme PAL was reduced ~8.0-fold in DPA and ~3.8-fold in BSO treated shoot tissues as compared to WT under infected condition (Fig. 4c).

### Development of susceptibility

**Fig. 5:**
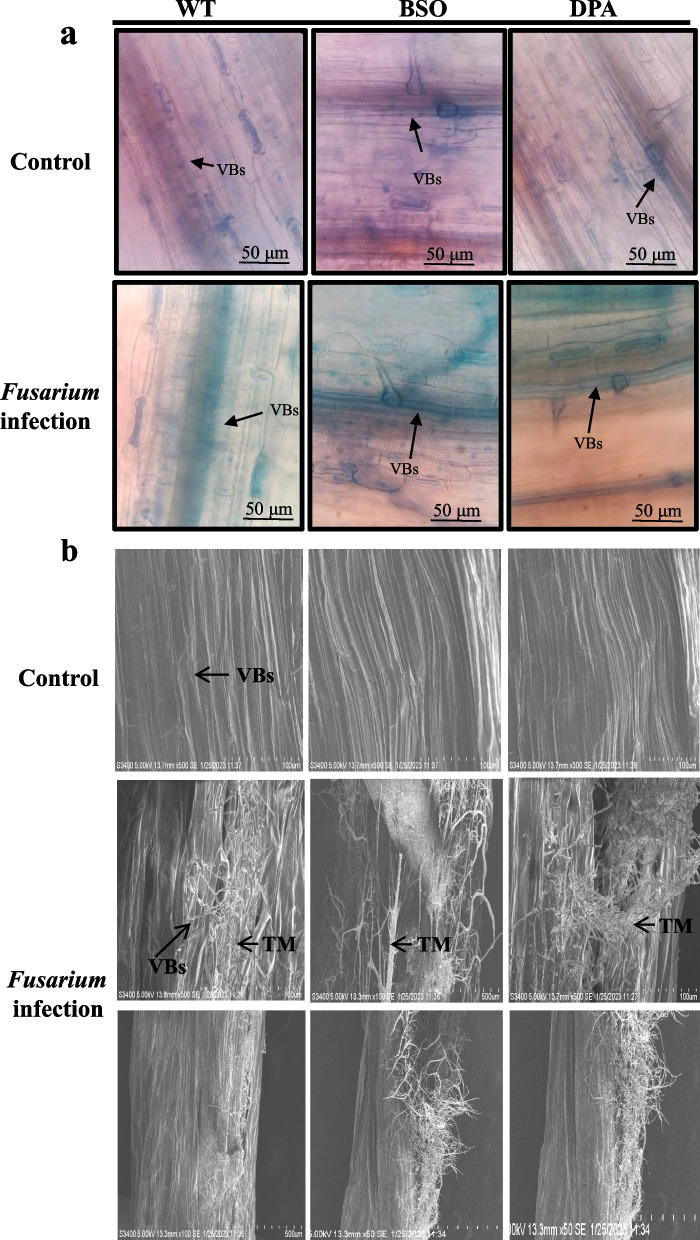
Detection of fungal structure. **a** Fungal development in leaf tissues, and **b** Fungal structure in VBs and damaged shoot tissues. VBs-Vascular bundles, TM-Thick mycelium

The BSO and DPA treated seedlings exhibited disease lesions 2 d after inoculation, whereas WT seedlings showed visible symptoms after 4 d (Fig. S1). The visual observation of fungal structures was performed by histochemical staining of leaves with lactophenol cotton blue (LPCB). The formation of dark blue spots indicative of fungal structure accumulation increased in the VBs of BSO and DPA treated leaf tissues but less in WT under infection condition (Fig. 5a). In the shoot tissues, the thick and branched mycelial network appeared more in VBs of BSO and DPA than in WT under infection condition. In addition, the disorganized xylem vessels were found more in BSO and DPA shoot tissues than WT. The proliferated mycelia exhibited more damaged tissues in BSO and DPA than WT shoot tissues (Fig. 5b).

**Fig. 6:**
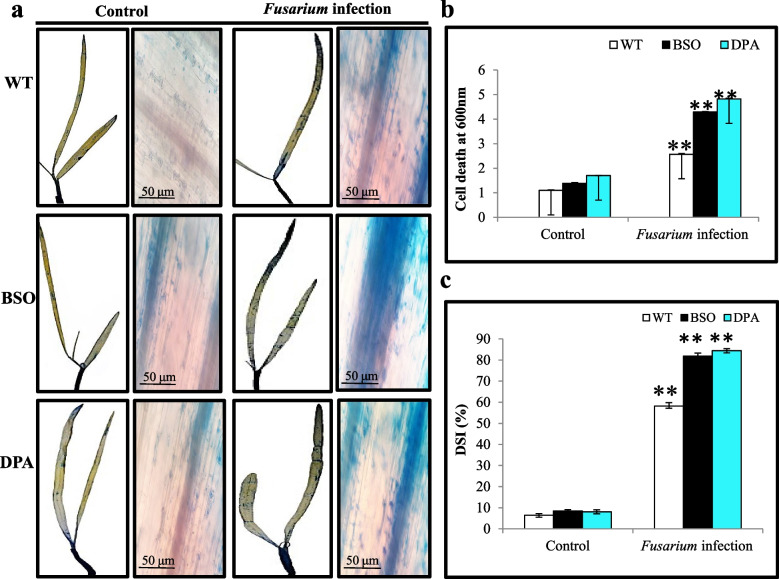
Evaluation of the effect of inhibitors on cell death analysis in WT, BSO, and DPA treated seedlings during *Fusarium* stress. **a** Histochemical assessment of Cell death in leaf tissues, **b** Cell death content in shoot tissues, and **c** Percentage (%) of DSI in seedlings. *, ** denote significance at *p* ≤ 0.05 and *p* ≤ 0.001, respectively

The BSO and DPA treated leaf tissues showed increased cell death, indicated by a darker blue color than WT in infection condition (Fig. 6a). Similarly, the BSO and DPA treated shoot tissues showed ~2.04 and ~2.27-fold more cell death content under infection condition than WT shoot tissues (Fig. 6b). The BSO and DPA treated seedlings showed an enhanced ~1.45 and ~1.54-fold Disease severity index (DSI) than WT during *Fusarium* infection (Fig. 6c). These results indicated that the application of 1 mM concentrations of BSO and DPA induces the growth and spreading mycelial network and increases the disintegration of shoot tissues.

**Fig. 7:**
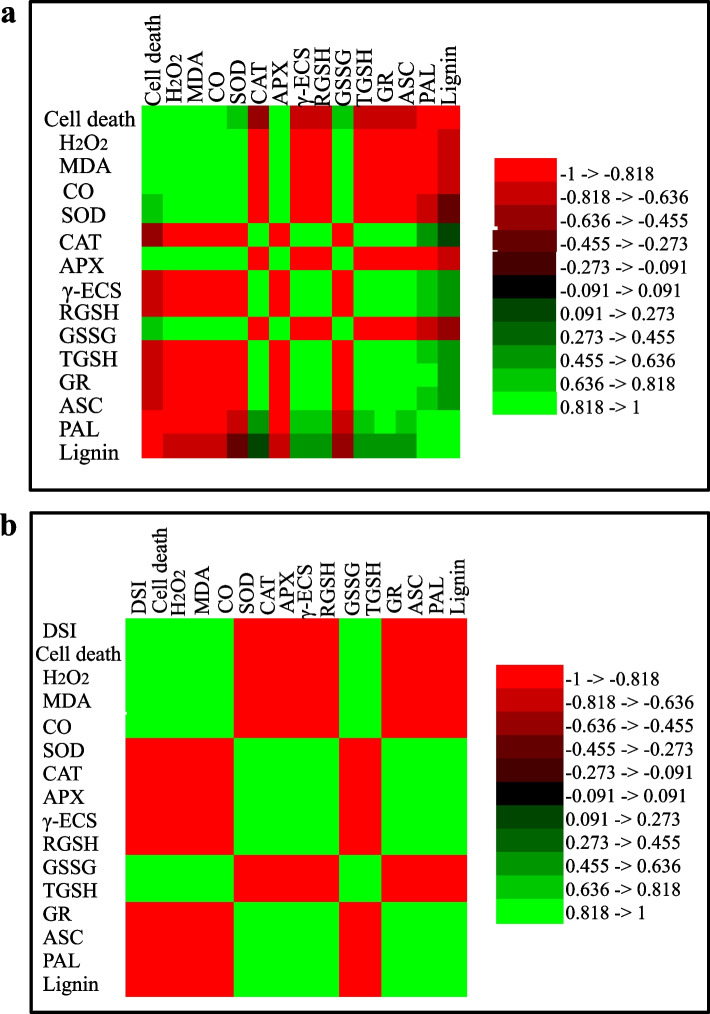
Correlation coefficient analysis showing the relationship among various parameters between control and *Fusarium* infection

The association between several factors in WT, BSO, and DPA treated plants was determined by analysing the correlation coefficient. The correlation coefficients for cell death, H_2_O_2_, MDA, SOD, CAT, APX, γ-ECS, GSH, GSSG, TGSH, GR, ASC, PAL, and Lignin for control and *Fusarium* infection are shown in Table S1 and Fig. 7. Significant correlations were observed between H_2_O_2_-DSI, H_2_O_2_-SOD, H_2_O_2_-APX, H_2_O_2_-Lignin, CO-MDA, γ-ECS-GSH, GSH-ASC, GR-GSSG, PAL-Lignin, and Lignin-DSI after BSO and DPA treatment (*P* ≤ 0.05) during infection. However, non-significant relationships were found between MDA and CO, γ-ECS, GSH, GSSG, and ASC at *P* ≤ 0.05.

## Discussion

Wheat, an economically significant agricultural plant, has previously been shown to acquire susceptibility to a variety of distinct fungal diseases, with *F. oxysporum* being one of the most prominent plant pathogens responsible for massive crop losses due to vascular wilt, yellows, corm rot, root rot, and damping-off diseases. The findings of the present study reveal that wheat seedlings develop a systemically induced susceptibility to *F. oxysporum* infection in response to BSO and DPA applications. Earlier results reported that inhibitors like 2,4-DPA and cinnamaldehyde (CAld) for PAL (Fujita et al. 2006), 2,4-dinitrophenol (DNP), MDCA, N-(3,4-dichlorophenyl)propanamide (propanil), and menadione (MD) for cinnamate 4-hydroxylase (C4H), caffeic acid (CA), and ferulic acid (FA) for 4-coumarate: CoA ligase (4CL) are used in the phenylpropanoid pathway to induce susceptibility in plants (Harding et al. 2002). For the formulation of effective ways to manage wheat susceptibility, it is necessary to explore and uncover the molecular mechanisms underlying plant immunity.

It has been established that under stressful condition, the physiological parameters like SL, RL, FW, DW, and RWC are disturbed and reduced the plants’ growth. In this study, treatment with BSO and DPA showed more reduced physiological parameters than WT under infected conditions. Similarly, the 2,4-DPA application reduces plant growth by increasing oxidative damage in the ZJ 88 (salt-sensitive) rice cultivar (Islam et al. 2017), and BSO also affects physiological aspects in *Arabidopsis thaliana* (Wójcik et al. 2009). Hence, the BSO and DPA treated seedlings showed higher chlorosis and severe wilting symptoms than the WT during infection. Moreover, the proliferation of *F. oxysporum* mycelium might inhibit water transportation, which resulted in wilt symptoms in BSO and DPA treated seedlings more quickly than in WT (Yadeta and Thomma 2013). Hence, the low RWC leads to dissociation of primary metabolites that supply intermediates to the defence system and ultimately leads to susceptibility to *F. oxysporum* (Bispo et al. 2016).

In pathogen attack, a quick formation and deposition of ROS arises, which is extremely hazardous to macromolecules and causes lipid peroxidation (MDA), protein carbonyl (CO) (an irreversible protein oxidation product) (Zhang et al. 2021), resulting disease progression in plant tissues (Bi et al. 2021), which is concomitant to the current study, where H_2_O_2_, MDA, and CO content were observed high in WT during *Fusarium* infection. Moreover, the high H_2_O_2_ was indicative of high susceptibility in BSO and DPA tissues, is evidenced by the localization of H_2_O_2_ in shoot tissues. Similarly, MAPK inhibitor (U0126) treatment increased H_2_O_2_ and MDA in plants infected with *Botrytis cinerea* (Zheng et al. 2015), resulting in cellular dysfunction and/or cell death (Lu et al. 2021). In addition, 3AB, a PARP inhibitor, also induced oxidation in *A. thaliana* (Briggs et al. 2017). Some earlier reports also stated that inhibitors like diphenylene iodonium (DPI), imidazole, tiron, and dimethylthiourea (DMTU) induced resistance in Arabidopsis by inhibiting the NADH oxidase enzyme and reducing ROS production (Wang et al. 2019).

SOD, CAT, and APX all play a part in eliminating ROS and regulating the cellular redox balance (Hojati et al. 2017; Islam et al. 2022). Many researchers have found that the antioxidant enzymes SOD, CAT, APX, and POD activity decreased during pathogenesis in immunocompromised mice (Łanocha-Arendarczyk et al. 2018; Kot et al. 2020). Here, a concomitantly low activity of CAT, APX, and SOD was observed in BSO and DPA treated tissues than WT under infection conditions, which augments susceptibility like U0126-treated tomato plants against *B. cinerea* infection (Wu et al. 2021). In addition, diethyldithiocarbamate (DDC) and 3-amino-1,2,4-triazole (AT) inhibitors were reported to reduce the activity of SOD and CAT, respectively, in rice seedlings (Chen et al. 2015). Moreover, the GR converts GSSG into RGSH and maintains the GSH pool in the plant (Jung et al. 2019). Both the BSO and DPA treated shoot tissues showed lower GR activity than WT during infection, as also shown by Raja et al. (2020) in *Solanum lycopersicum* during drought, heat, and salinity stress conditions. Thus, the decreased antioxidant activity of the treated plants helps to overexpress ROS levels and their effect on membranes (MDA and CO formation).

ASC and GSH are both involved in the crucial ASC-GSH cycle, which aids in the detoxification of H_2_O_2_ (Noctor et al. 2018) and is regulated by APX, DHAR (dehydroascorbate reductase), MDHAR (monodehydroascorbate reductase), and GR (Hasanuzzaman et al. 2019). Here, the BSO treated shoot tissues showed lower GSH content than DPA and WT during infection, which would be the consequence of irreversible inhibition of the γ-ECS enzyme by the redox modulator BSO (Banerjee et al. 2018; Sehar et al. 2021). Moreover, reduced GSSG content was found in BSO treated shoot tissues than DPA and WT during infection, due to inhibition of GSH synthesis (Sehar et al. 2021). The DPA treated showed higher GSSG than WT, which was indicative of elevated H_2_O_2_ induced GSH oxidation (Chen et al. 2020) in *Pisum sativum* (Romero-Puertas et al. 2004). In addition, the TGSH was observed to be lower in BSO treated shoot tissues than in DPA and WT due to the oxidative burst induced by plant-pathogen interactions (Matern et al. 2015). The BSO and DPA treated shoot tissues could not regenerate the ASC due to lower activity of APX during infection conditions (Hossain et al. 2022) than WT, resulting in a disrupted ASC-GSH cycle that makes them susceptible to pathogens (Hernández et al. 2017; Schlaeppi et al. 2008). In addition, the γ-ECS is the rate-limiting step for the overall GSH biosynthesis. Furthermore, the γ-ECS-deficient plants showed a low level of GSH, rendering them susceptible to pathogens (Noctor et al. 2012; Hiruma et al. 2013), which is consistent with our result that BSO treated shoot tissues declined γ-ECS activity and RGSH more than WT during infection (Thompson et al. 2021). In addition, the DPA treated shoot tissues also reduced γ-ECS activity and RGSH (Romero-Puertas et al. 2004). The γ-ECS enzyme activity was lowered in plants during *Trypanosoma cruzi* infection (Vázquez et al. 2017), which is similar in WT plants. Moreover, the lipoxygenase inhibitor ibuprofen (IBU) suppressed the TGSH and γ-ECS activity in *Agropyron cristatum* leaves (Shan and Liang 2010). The pattern of the ASC-GSH cycle is also reduced as marginally in BSO and DPA treated shoot tissues than in WT under the control condition but reduced highly in the infection condition, which might be an influence of *F. oxysporum* pathogenicity.

The lignification machinery has been linked with the basal immunity of *A. thaliana* during fungal interaction (Cesarino 2019). The decline of lignin deposition was observed in VBs of DPA and BSO treated shoot tissues compared to WT during infection and was also corroborated by lignin content. Similarly, the use of PAL inhibitors AIP and AOPP (100 µM) inhibited lignin accumulation in tracheary elements (Dennis et al. 2004). PAL is the key enzyme for lignification barriers to fungal invasion into the cell walls of VBs (Cass et al. 2015) and was shown to be regulated by the herbicide 2,4-DPA in potato plants (Nassar and Adss 2016). Treatment of seedlings with AOPP (Pan et al. 2008), 1.0 mM 2,4-DPA, and 0.5 M acetic acid (Tomás-Barberán et al. 1997), a chemical inhibitor of PAL activity, reduced the accumulation of lignin content. In addition, *F. virguliforme* also reduced lignin deposition in controlled soybean plants (Giachero et al. 2022). PAL activity was decreased in DPA and BSO than in WT treated shoot tissues during infection, indicating the declination of lignin in VBs. The depleted lignin can also promote the proliferation of *Fusarium* mycelium in xylem tissues (El-Ganainy et al. 2023). Hence, both BSO and DPA induce susceptibility through the reduction of lignin deposition and PAL activity in WT against *F. oxysporum*, similar to the poly(ADP-Ribose) polymerase (PARP) enzyme’s inhibitor,3-methoxybenzmide (3AB) in Arabidopsis against *Botrytis cinerea* (Adams-Phillips et al. 2010).

*F. oxysporum* localization directly reflects the degree of development of disease symptoms; these measures are widely used in the study of plant susceptibility responses. *F. oxysporum* spores germinated and developed into mycelia in the xylem vessels, which further entered the cortex and VBs to transform into thick mycelia and cause disintegration at the tissue level, as also reported by Banerjee et al. (2018). Finally, the disintegrated VBs degrade the central pith and other tissue parts, causing high vascular wilt in BSO and DPA. Zhang et al. (2015) also reported that *F. oxysporum* exhibited disintegration of the xylem, collapse of the parenchyma tissues, and digestion of the central pith in watermelon seedlings. Moreover, the infected hyphae proliferated throughout the BSO and DPA treated shoot, resulting in enhanced damaged tissues, indicating the high susceptibility by BSO and DPA, like AIP, AOPP (Dennis et al. 2004), and IBU (Shan and Liang 2010).

The pathogen *Cochliobolus victoriae* induces cell death in immunocompromised Arabidopsis and oats (Kessler et al. 2020), which was consistent with our study that *F. oxysporum* caused cell death in immunocompromised WT induced by BSO and DPA. The elevated cell death induces susceptibility in the host, which indicates successful pathogenesis (Coll et al. 2011). Similarly, 1-methylcyclopropane (1-MCP) inhibited ethylene receptors in red winter wheat and induced susceptibility during heat stress (Hays et al. 2007). Aminoxyacetic acid (AOA), aminoethoxyvinylglycine (AVG), and aminohydrazinophenylpropionic acid (AHPP) reduce the activity of aminotransferase and PAL, which promotes susceptibility in the NC2326 cultivar of tobacco plant (Saindrenan and Guest 2017). The high degree of fungal colonization of *F. oxysporum* and DSI also confirmed the susceptibility of BSO and DPA treated plants as compared to the WT, which is consistent with study by Kocsy et al. (2000).

The significant correlations between H_2_O_2_-DSI, H_2_O_2_-SOD, H_2_O_2_-APX, and H_2_O_2_-lignin may be due to *Fusarium* induced oxidative stress (Samsatly et al. 2018). The close relationship between γ-ECS-GSH, GSH-ASC, and GR-GSSG may possibly be attributed to the fact that γ-ECS is the key regulator of the ASC-GSH cycle (Hiruma et al. 2013). The lignin-DSI correlation might be due to the depletion of lignin content through the inactivation of PAL.

## Conclusions

**Fig. 8:**
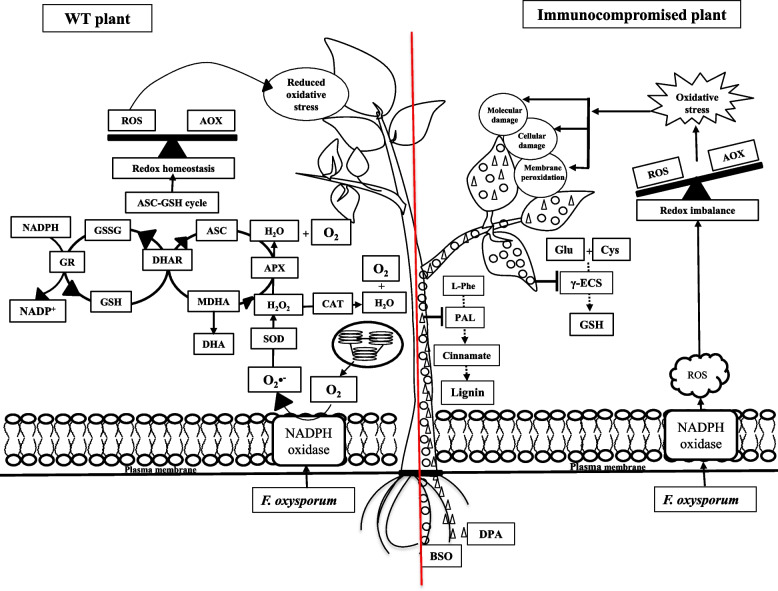
The schematic representation of inhibitors induced susceptibility in WT seedlings during *Fusarium* infection. The inhibitors (BSO and DPA) inhibit the activity of γ-ECS and PAL, respectively which serve as key players in the defence system against oxidative stress. Hence, induces susceptibility in plant against *F. oxysporum*. *Abbreviations*: BSO-L-Buthionine-sulfoximine; 2,4-DPA-2,4-dichlorophenoxy acetic acid; O_2_**•**^**−**^-Superoxide anion; OH•-Hydroxyl radical; 2O_2_•^−^-Peroxide; O_2_-Oxygen; H_2_O_2_-Hydrogen peroxide; 2H^+^-Hydrogen ion; H_2_O-Water; MDA-Malondialdehyde; CO-Carbonyl; SOD-Superoxide dismutase; CAT-Catalase; APX-Ascorbate peroxidase; GR-Glutathione reductase; RGSH-Reduced glutathione; TGSH-Total glutathione; ASC-Ascorbate; γ-ECS-γ-glutamyl cysteine synthetase; GSSG-Oxidized glutathione; L-Phe-L-phenylalanine; PAL-Phenylalanine ammonia-lyase *Symbols:* BSO(○), DPA(Δ), Inhibition(┤), Synthesis of product (─), Reduction of product(---)

In conclusion, 1 mM BSO and DPA treatment significantly inhibited the activity of γ-ECS and PAL, which resulted in an increase in susceptibility against *F. oxysporum*. A schematic diagram of inhibitors induced susceptibility has been represented in Fig. 8. Thus, the increase in susceptibility caused by inhibitors may be due to the decrease in GSH and lignin content, which serve as key players in the defence system against fungal pathogens.

## Materials and methods

### Plant material and growth conditions

Healthy and viable wheat seeds (*T. aestivum* Var. Sharbati Sonora) were surface sterilized using 0.01% HgCl_2_, followed by three times washing with sterile distilled water. In order to promote germination, the sterilized seeds were kept in a sterile beaker containing distilled water and incubated for 48 h at 26 °C in the dark period. Following incubation, the seeds were again cleaned with distilled water before being placed in a sterilized Petri dish lined with a wet muslin cloth. Then, the plates were incubated at room temperature (RT) for 4–5 days (d). Young seedlings that had sprouted with uniform root and shoot growth were put into sterile glass test tubes filled with distilled water and kept in a growth chamber for 7 d at 32 °C,80% relative humidity, with a 16 h photoperiod (240 µmol/ m^2^s) and 8 h dark period at 26 °C, 70% relative humidity (Mittra et al. 2004).

### Inhibitor treatments and pathogen inoculations

*F. oxysporum* (FMU01) was grown under dark and immobilized conditions at 28 ± 2 °C for 4 d to produce efficient sporulation. Sprouted wheat saplings were transported to sterilized glass test tubes containing distilled water after 7 d. One set of seedlings was incubated with 1 ml of 1 mM BSO and another set with 1 ml of 1 mM 2,4-DPA suspension for 48 h at RT (Flores-Cáceres et al. 2015). In a control set, seedlings were grown in DW and kept under the same conditions. After that, the wheat roots were inoculated with 4 d old *F. oxysporum* spores (1 × 10^5^ spores ml^−1^) by pouring the spore culture into each set of test tubes and kept for another 7 d for further growth and development.

### Plant growth parameters

After 7 DAI (day after inoculation), the seedlings from each experimental setup were taken for determination of shoot length (SL), root length (RL), fresh weight (FW), dry weight (DW), and relative water content (RWC). The RWC was calculated using the method and followed the below equation (Tahjib-Ul-Arif et al. 2018).

$$\mathrm{RWC}(\mathrm{\%})=\frac{(\mathrm{Fresh weight}-\mathrm{Dry weight})\times 100}{(\mathrm{Turgor weight}-\mathrm{Dry weight})}$$

### Measurement of oxidants

#### In planta histochemical detection of H_2_O_2_

The localization of H_2_O_2_ was carried out histochemically in seedlings using the method described by Daudi and O’Brien (2012). The seedlings were dipped in sterilized glass beaker containing 3,3′-Diaminobenzidine (DAB) solution (1 mg ml^−1^, pH 4.0) for 12 h under the light at RT. The seedlings were then dipped in 95% ethanol and boiled for 20 min to decolorize them. After the decolorization,the localized H_2_O_2_ was visualized as brown spots.

#### Assay of H_2_O_2_ content

The H_2_O_2_ content was measured through the protocol of Noreen et al. (2009). Fresh shoot tissues were homogenized with 0.1% trichloroacetic acid (TCA), for supernatant collection through centrifugation at 12,000 rpm for 15 min. The reaction mixture containing 0.5 ml of the supernatant, 0.5 ml of 10 mM phosphate buffer (pH 7.0) and 1 ml of 1 M KI in a cuvette was measured at a 390 nm wavelength. The H_2_O_2_ content was calculated by the molar extinction co-efficient 0.28 µM^−1^ cm^−1^ and was expressed as µM g^−1^ f.w.

#### Assay of MDA content

MDA was assayed using the method described by Basu et al. (2010). 0.5 ml of 5% Trichloroacetic acid (TCA) was used to homogenise the shoot tissues and centrifuged at 12,000 rpm for 20 min. For MDA estimation, 2 ml of thiobarbituric acid (TBA) reagent (0.5% TBA in 20% TCA) was mixed with 0.5 ml of supernatants. The absorption of MDA-TBA abduct was evaluated by the molar extinction co-efficient 155 mM^−1^ cm^−1^ at 532 nm, and nonspecific turbidity was corrected by subtracting absorbance at 600 nm, expressed as µM g^−1^f.w.

#### Assay of protein-carbonyl content

The protein carbonyl content was estimated by Basu et al. (2010). The purified proteins were precipitated in 0.5 ml of 15% TCA under cold condition for 15 min, then the precipitants were centrifuged at 10,000 rpm. The protein pellets were washed with 20% TCA for two times. The final protein pellet was redissolved in 0.5 ml of 0.2 mM sodium phosphate buffer (pH 7.0). The absorbance of protein-carbonyl content was estimated at 360 nm using the molar extinction coefficient of 2,4-Dinitrophenylhydrazine(DNP) (17,530 µM^−1^ cm^−1^), and expressed in µM g^−1^f.w.

### Measurement of the activities of antioxidant enzymes

#### Assay of SOD activity

The SOD activity was assayed by photo-inhibition of nitro blue tetrazolium (NBT) at 560 nm using the molar extinction coefficient of 12.8 L mol^−1^ cm^−1^, by Kumari et al. (2015). The 3 ml reaction mixture containing 50 mM phosphate buffer (pH 7.8), 0.3 ml of 20 µM riboflavin, 0.3 ml of 130 mM methionine, 0.3 ml of 750 µM NBT, 0.3 ml of 10 mM EDTA, 0.25 ml of distilled water, and 50 µl extracted enzymes was taken in sterilized test tubes and placed under a fluorescent lamp for 10 min. The 1 unit (U) of SOD activity is considered the quantity of enzyme required to cause 50% inhibition of the reduction of NBT.

$$\mathrm{\%\, of\, inhibition}= [1-\mathrm{ Absorbance\, of\, each\, sample\, }/\mathrm{\, Absorbance\, of\, the\, control}] \times 100$$

#### Assay of CAT activity

The CAT activity was determined by Zhang et al. (2021). The reaction mixture containing 50 µl of 30 mM H_2_O_2_, 2.9 ml of 50 mM enzyme extract was taken in a cuvette. The decreased absorbance was estimated at 240 nm for 3 min using the molar extinction coefficient 40 mM^−1^ cm^−1^ and expressed in U g^−1^f.w.

#### Assay of APX activity

The APX activity was measured using the molar extinction coefficient of 2.8 mM^−1^ cm^−1^ for the ASC method by Kumari et al. (2015). 1 ml of the reaction mixture comprising 600 µl of 50 mM phosphate buffer solution, 100 µl of 1 mM EDTA, 100 µl of 5 mM ascorbic acid, 100 µl of H_2_O_2_, and 100 µl of the enzyme extracts were taken in a cuvette. The reduced absorbance was reported at 290 nm and expressed in U mg^−1^ protein. 1U of APX activity is established as the quantity of enzyme needed to decrease 1 µmol of H_2_O_2_ min^−1^ under the assay condition.

#### Assay of GR activity

According to the GR assay, the homogenates were prepared using 50 mM Tris-HCl buffer (pH 7.5), contained 1 mM EDTA, 9.94 mM sodium ascorbate, and 0.5% insoluble polyvinylpyrrolidone. Then the homogenates were centrifuged at 12,000 rpm for 20 min. The 3 ml reaction mixture containing 50 mM Tris-HCl (pH 7.5), 3 mM MgCl_2_, 1 mM GSSG, 0.2 mM EDTA, and 0.3 ml enzyme extracts were taken into a cuvette. The enzyme activity was estimated at 340 nm for 1 min using the molar extinction coefficient of 6.22 × 10^3^ M^−1^ cm^−1^ and expressed as U g^−1^ f.w. following the method of Sahoo et al. (2019).

### Measurement of antioxidants and metabolite

#### Assay of total GSH (TGSH) content

The contents of RGSH and GSSG were estimated by the 5,5’-dithiobis-2-nitrobenzoic acid (DTNB)-GSSG reductase method outlined by Ogawa et al. (2004). The rate of formation of 5-thio-2-nitrobenzoate (TNB) was measured at 412 nm using the molar extinction coefficient of 0.017 mM^−1^ cm^−1^ and GSH was taken as a reference. The 4-polyvinylpyrrolidone was used to trap the GSH present in the 5-sulfosalicylic acid supernatant solution (2 µl/100 µl).

#### Assay of ASC content

The ASC content was measured by the following method by Ainsworth and Gillespie (2007). The 0.5 ml of charcoal treated enzyme extracts, 1.5 ml of 4% TCA, 0.5 ml of 2% dinitrophenylhydrazine (DNPH), and 2 drops of 10% thiourea solution were combined and incubated at 37 °C for 3 h to generate osazone crystals. The crystals were dissolved in 85% H_2_SO_4_ under cold conditions, and the absorption was read at 540 nm using the molar extinction coefficient of 2.88 mM^−1^ cm^−1^, and expressed in µg g^−1^f.w.

#### Assay of γ-ECS activity

The *γ-*ECS activity was measured by following the method of Ogawa et al. (2004). The 1 ml reaction mixture consisting of 500 µl of enzyme extract and 50 mM Tris–HCl (pH 7.6) contained 1 mM dithioerythritol, 10 mM ATP, 0.25 mM glutamate, and 2 mM cysteine and was incubated at 25 °C for 1 h. Then, the reaction mixture was mixed with 1.2 ml of phosphorous agent, which contained 2.5% ammonium molybdate, 10% vitamin C, and 3 mM H_2_SO_4_ followed by incubation at 45 °C for 25 min. The mixture was taken for absorbance at 660 nm using the molar extinction coefficient 125 M^−1^ cm^−1^ and expressed as U mg^−1^ protein.

#### In planta histochemical detection of lignin

The accumulated lignin was histochemically stained with phloroglucinol following the method by Veronico et al. (2018). The shoot tissues were immersed in 70% ethanol for 2 min, followed by treatment with HCl for 1 min. The lignin was visualized as a red-orange colour under a light microscope.

#### Lignin content

The lignin content was assayed using the protocol of Sharma et al. (2000). The dried methanol extracts contained 50 mg of alcohol insoluble residues, 0.5 ml of TGA, and 5 ml of 2 N HCL. They were boiled for 4 h. Then, the mixture was suspended in 5 ml of 0.5 N NaOH, followed by the addition of 1 ml of HCL to precipitate the lignin-TGA compound. The lignin-TGA compound was estimated at 280 nm and expressed in A_280_ g^−1^ of alcohol insoluble residues f.w.

#### Assay of PAL activity

PAL activity was assayed using the molar extinction coefficient of 10.238 M^−1^ cm^−1^ by Umesha (2006). 1 ml of plant enzyme extract was mixed with 0.5 ml of 50 mM L-phe and 0.4 ml of 25 mM borate buffer in setrilized test tubes. The tubes were incubated for 2 h at 400 °C in a water bath. To stop the reaction, 0.06 ml of 5 N HCL was added in tubes to read the absorbance at 290 nm against L-phe as a blank, expressed as moles of transcinnamic acid m^−1^ mg^−1^ protein.

### Detection of fungal colonies in infected seedlings

A mixture of acetic acid, ethanol, and water (2:2:1, v/v/v) was used to decolorize the shoot pieces at 25 °C to determine fungal colonies by Garg et al. (2010). Then, the parts were washed in deionized water and stained with a 1% lactophenol cotton blue solution to image the fungal colonies in blue.

Two millimeter (2-mm) long shoot sections were cut using a sterile scalpel under aseptic conditions. The shoot parts were fixed by immersing in 5% glutaraldehyde in 0.1 M phosphate buffer (pH 7.2) for 4 h. Then the tissue parts were dehydrated by passing them through a series of aqueous ethanol solutions (10, 30, 50, 75, and 95%) and then placed in 100% ethanol, each for 5 min at RT. The tissue parts were dried, mounted on aluminium stubs, and coated with gold film in a sputter coater for 15 min. The tissue segments were observed under a scanning electron microscope (SEM) (Hitachi, S3400N, 30 kV).

### Estimation of cell death and disease severity index (DSI)

The dead cells were examined by using trypan blue following the method of Kerschbaum et al. (2021). The seedlings were dipped into 40 ml of 0.01 g of trypan blue for 1 min at RT. Then, the seedlings were cleaned with a washing solution containing ethanol and water (1:1) to visualise the polymerized blue colour as dead cells.

Cell death was assayed by Gölge and Vardar (2020). The shoot tissues were dipped in 10 ml sterilized test tubes containing 1 ml of 0.25% Evans blue, and the aliquot was measured at 600 nm and expressed in percentage (%).

Using a (0–3) intensity scale, the DSI of wheat plants was evaluated after 7 DAI (Strelkov et al. 2006). According to the morphology of the root organs, the seedlings were divided into four groups.

Healthy plants 0 = No root rot symptoms

Slightly infected plants 1 = Dark brown to black spots on root

Healthy infected plants 2 = Weak, stunted, and rotting rot seedlings

Dead plants 3 = Dead and fallen seedlings

The following equation was used to measure the DSI.

$$\mathrm{DSI}(\mathrm{\%})= \frac{\sum [(\mathrm{Class\, Number})(\mathrm{Number\, of\, plants\,in\, each\, class})]}{\left(\mathrm{Total\, number\, of\, plants\,per\, sample}\right)(\mathrm{Number\, of\, classes}-1)}\times 100$$

### Data analysis by correlation coefficient

For various redox parameters of the WT, BSO, and DPA seedlings, values are presented as the mean of three replicates. Here, the mean of three replicates represents the “mean of three independent seedlings”. The results were assessed by the Student’s t-test. Significance was defined as *p* ≤ 0.05 (*) and *p* ≤ 0.001 (**). The covariance correlation was carried out using XLSTAT, 2020 software (XLSTAT, Addinsoft, New York, NY).

### Supplementary Information

**Additional file 1: Table S1.** Pearson’s correlations (R) between the parameters like H_2_O_2_, MDA, CO content, antioxidant enzyme activities, antioxidants, and lignin in WT, BSO, and DPA treated seedlings during *Fusarium* stress.* Abbreviations*: WT-Wild type; BSO- L-Buthionine-sulfoximine; 2,4-DPA-2,4-dichlorophenoxy acetic acid.

**Additional file 2: Fig. S1.** Progress of disease with time. The appearance of disease symptoms on leaves of a diseased seedlings from day 2 to day 7. *Abbreviations*: WT-Wild type; BSO- L-Buthionine-sulfoximine; 2,4-DPA-2,4-dichlorophenoxy acetic acid.

## Data Availability

Not applicable.

## Abbreviations

15ADON: 15-Acetyl deoxynivalenol
1-MCP: 1-Methylcyclopropane
^1^O_2_: Singlet oxygen
3AB: 3-Methoxybenzmide
3ADON: 3-Acetyl deoxynivalenol
4ANIV: 4-Acetyl nivalenol
4CL: 4-Coumarate: CoA ligase
4-HNE: 4-Oxo-2-nonenal
ACR: Acrolein
AHPP: Aminohydrazinophenylpropionic acid
AIP: 2-Aminoindane-2-phosphonic acid
AOA: Aminoxyacetic acid
AOPP: (S)-2-aminooxy-3-phenylpropionic acid
APX: Ascorbate peroxidase
ASC: Ascorbate
AT: 3-Amino-1,2,4-triazole
AVG: Aminoethoxyvinylglycine
BSO: L-Buthionine-sulfoximine
C4H: Cinnamate 4-hydroxylase
CA: Caffeic acid
CAld: Cinnamaldehyde
CAT: Catalase
CO: Carbonyl
D: Days
DAI: Days after inoculation
DDC: Diethyldithiocarbamate
DHAR: Dehydroascorbatereductase
DMTU: Dimethylthiourea
DNP: 2,4-Dinitrophenol
DON: Deoxynivalenol
DPA: 2,4-Dichlorophenoxyacetic acid
DPI: Diphenylene iodonium
DSI: Disease severity index
DW: Dry weight
FA: Ferulic acid
FW: Fresh weight
GPx: Guaiacol-peroxidase
GR: Glutathione reductase
GS: Glutathione synthetase
GSH: Glutathione
GSSG: Oxidized glutathione
H_2_O_2_: Hydrogen peroxide
IBU: Ibuprofen
KCN: Potassium cyanide
LPCB: Lactophenol cotton blue
MD: Menadione
MDA: Malondialdehyde
MDCA: 3,4-Methylenedioxycinnamic acid
MDHAR: Monodehydroascorbatereductase
MeHg: Methylmercury
NIV: Nivalenol
NO•: Nitric oxide
O_2_•^−^: Superoxide
OBHA: O-benzylhydroxylamine
PAL: Phenylalanine ammonia-lyase
PARP: Poly(ADP-Ribose) polymerase
PIP: Piperonylic acid
PPO: Polyphenol oxidase
Propanil: N-(3,4-dichlorophenyl)propanamide
PUFAs: Polyunsaturated fatty acids
RCS: Reactive carbonyl species
RGSH: Reduced glutathione
RHS: Reactive halogen species
RL: Root length
RNS: Reactive nitrogen species
ROS: Reactive oxygen species
RS: Reactive species
RT: Room temperature
RWC: Relative water content
SEM: Scanning electron microscope
SL: Shoot length
SOD: Superoxide dismutase
TGSH: Total glutathione
VBs: Vascular bundles
WT: Wild-type
γ-ECS: γ-Glutamylcysteine synthetase
