# Supplementary material for: Immunocompromisation of wheat host by L-BSO and 2,4-DPA induces susceptibility to the fungal pathogen Fusarium oxysporum
Source: Stress Biol. 2024 Apr 9;4(1):21. doi: 10.1007/s44154-023-00137-7 (PMC11004106; doi:10.1007/s44154-023-00137-7)
Supplement: Supplementary file 2 — Additional file 2: Fig. S1. Progress of disease with time. The appearance of disease symptoms on leaves of a diseased seedlings from day 2 to day 7. Abbreviations: WT-Wild type; BSO- L-Buthionine-sulfoximine; 2,4-DPA-2,4-dichlorophenoxy acetic acid. [file 44154_2023_137_MOESM2_ESM.pptx]

## Slide 1
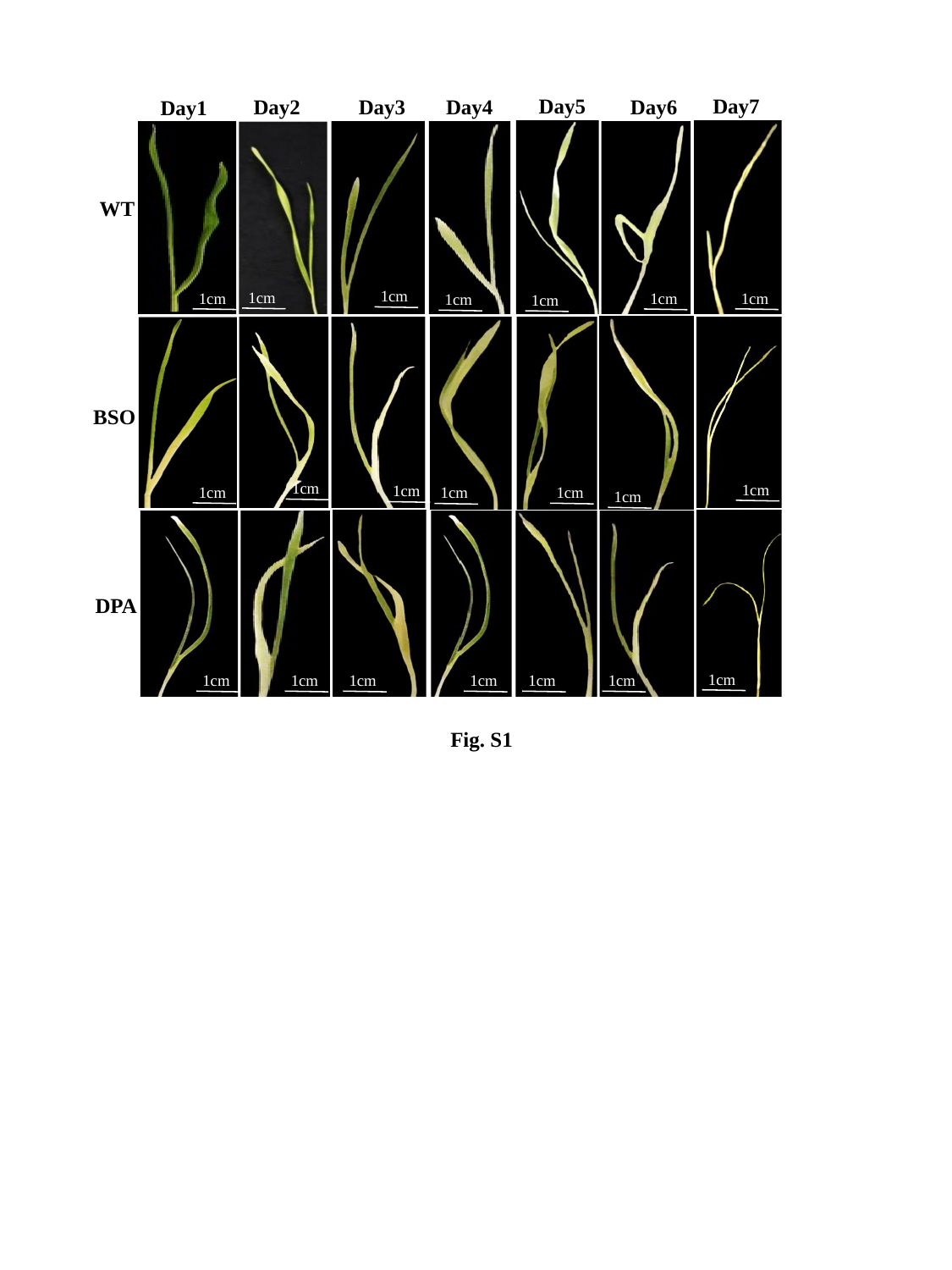

Day5
Day7
Day3
Day2
Day4
Day1
Day6
WT
BSO
DPA
Fig. S1
 1cm
 1cm
 1cm
 1cm
 1cm
 1cm
 1cm
 1cm
 1cm
 1cm
 1cm
 1cm
 1cm
 1cm
 1cm
 1cm
 1cm
 1cm
 1cm
 1cm
 1cm
